# Supplementary material for: AdRAP2.3, a Novel Ethylene Response Factor VII from Actinidia deliciosa, Enhances Waterlogging Resistance in Transgenic Tobacco through Improving Expression Levels of PDC and ADH Genes
Source: Int J Mol Sci. 2019 Mar 8;20(5):1189. doi: 10.3390/ijms20051189 (PMC6429156; doi:10.3390/ijms20051189)
Supplement: Supplementary file 1 [file ijms-20-01189-s001.pdf]

```
1       ATGTGCGGCGGTTCAATCCTCGACGAATTCATCCCTCGCAACGGTAACCACCGCGTCTCC
1        M  C  G  G  S  I  L  D  E  F  I  P  R  N  G  N  H  R  V  S
61      GCCTCCCAACTCTGGCCCAACTCCCCCTTCGTCACCAAATTCAAACCCCCACAAGATCAA
21       A  S  Q  L  W  P  N  S  P  F  V  T  K  F  K  P  P  Q  D  Q
121     AACGACGGTGATGAGCGTGTTGAAAAGAAGGCCAAGAGACAGCGCAAGAACCTGTACAGG
41       N  D  G  D  E  R  V  E  K  K  A  K  R  Q  R  K  N  L  Y  R
181     GGAATTAGGCAGCGTCCGTGGGGAAAATGGGCAGCGGAGATCCGAGATCCGAGAAAAGGT
61       G  I  R  Q  R  P  W  G  K  W  A  A  E  I  R  D  P  R  K  G
                                       *              *
241     GTGAGGGTTTGGATCGGTACCTTCAACACGGCCGAAGAGGCCGCCAGAGCCTACGACAGG
81       V  R  V  W  I  G  T  F  N  T  A  E  E  A  A  R  A  Y  D  R
301     GAAGCTCGCAAGATCCGAGGCAACAAAGCCAAGGTTAATTTTCCCAACGAAGACGACCAT
101      E  A  R  K  I  R  G  N  K  A  K  V  N  F  P  N  E  D  D  H
361     TCCATTCAATTTACTCCACAAACCCATCATTTACCCACCGCTATGAGTCATCCCAACGGA
121      S  I  Q  F  T  P  Q  T  H  H  L  P  T  A  M  S  H  P  N  G
421     GGGTTTAGTGGCAATCTGAACCAGTTTGGGGCATACAGCTCTAATGGGTTCAATAGCGTC
141      G  F  S  G  N  L  N  Q  F  G  A  Y  S  S  N  G  F  N  S  V
481     CCCTGTTCCGACCCTGTTTCGGTTCTTCACTTTGAAGAAATTTCTGGGTCTGGTTTAGAA
161      P  C  S  D  P  V  S  V  L  H  F  E  E  I  S  G  S  G  L  E
541     AGTTCTTACTCTTCGATTGATTTCAAATTAGAGGTGAAGGAAGAGAGAGAGAAGCAAGAG
181      S  S  Y  S  S  I  D  F  K  L  E  V  K  E  E  R  E  K  Q  E
601     GAGAGAGGAAACAGGAAGGAAGCGGCGGTGATGGAAGTGGAGGAAGCAGCAGGGGAAGAG
201      E  R  G  N  R  K  E  A  A  V  M  E  V  E  E  A  A  G  E  E
661     AGCGAAGTGGAGAAGCTGTCGGAGGAGTTGATGGCCTACGAGTCCGTCATGAAATTCTAT
221      S  E  V  E  K  L  S  E  E  L  M  A  Y  E  S  V  M  K  F  Y
721     CAGATTCCATATCTCGACGGCCAATCGACGAATGCTCCGCCAGCGGAGAACGACGTCATC
241      Q  I  P  Y  L  D  G  Q  S  T  N  A  P  P  A  E  N  D  V  I
781     GGCTGTGGTGCTGTGGAACTATGGAGCTTCGACGATCTTACCCCTACGGTGGCCTGA
261      G  C  G  A  V  E  L  W  S  F  D  D  L  T  P  T  V  A  *
```

Figure S1. Nucleotide sequence and deduced amino acid of *AdRAP2.3* gene from *A. deliciosa*. The AP2/ERF conserved domain is underlined, the conserved 14th alanine and 19th aspartic acid are marked with asterisks.

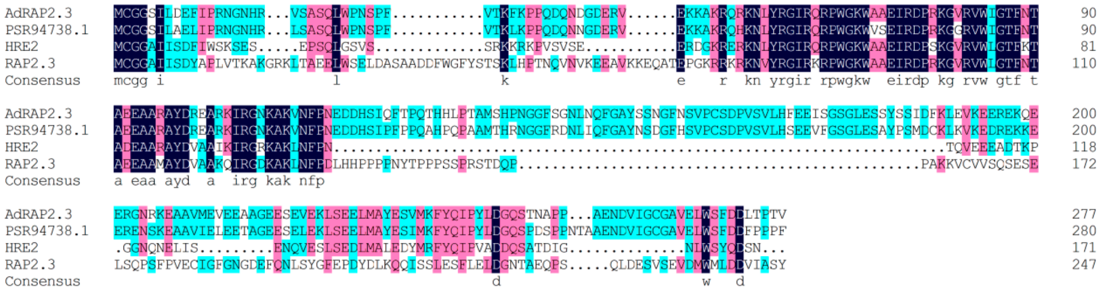

Figure S2. Protein sequence alignment of AdRAP2.3 with the PSR94738.1, HRE1, RAP2.3 proteins.

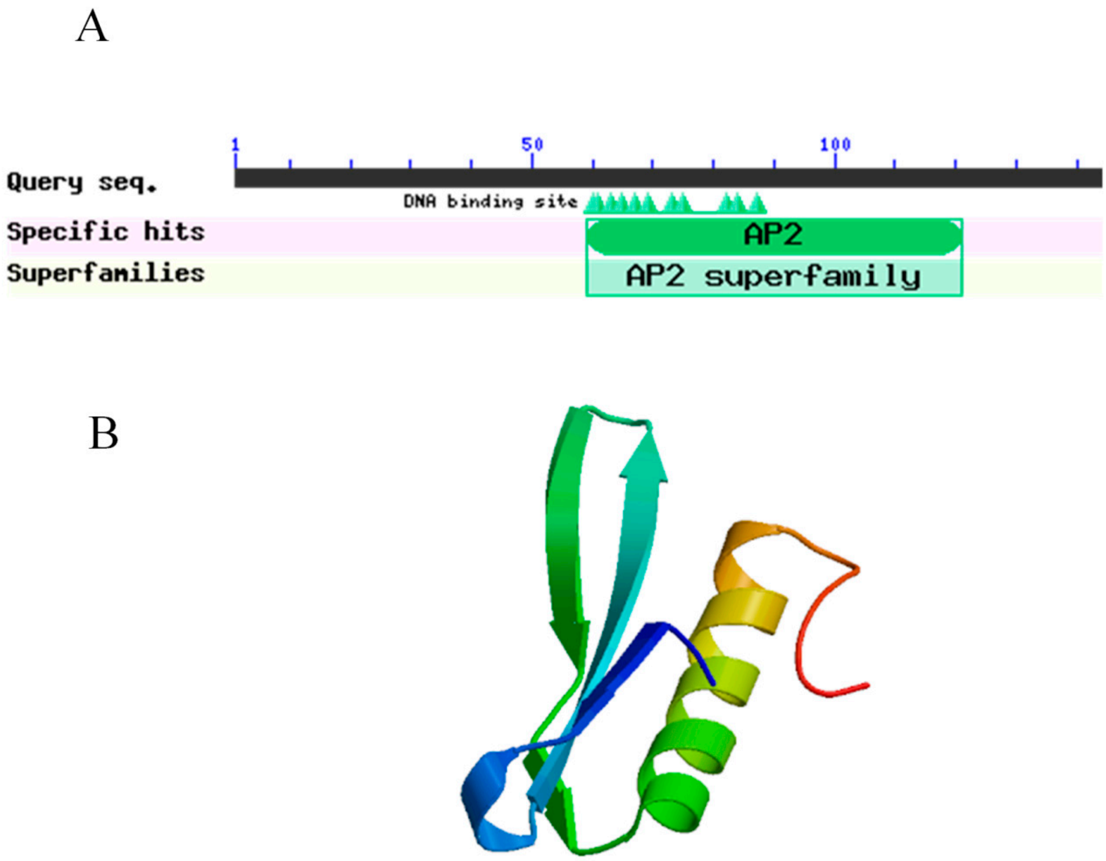

Figure S1. Secondary and three-dimensional structure of main part of AdRAP2.3

A: AP2 superfamily and DNA binding site; B: one α-helix and three β-flods

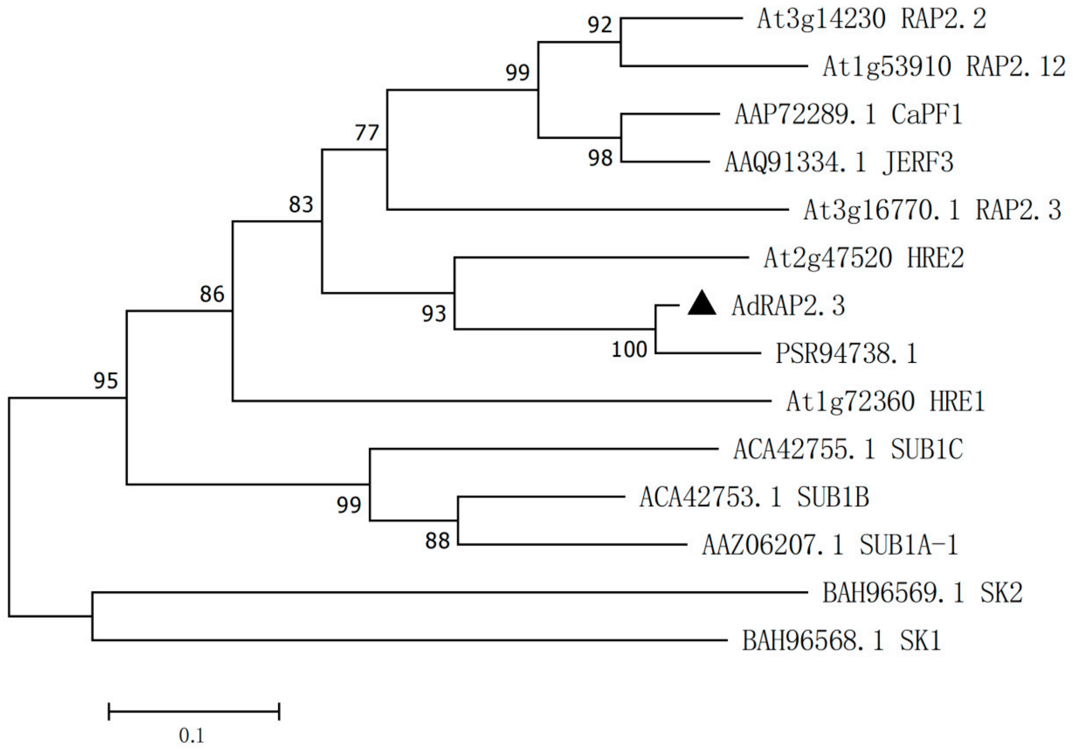

Figure S4. Neighbor-joining phylogenetic analysis of AdRAP2.3 with other ERF VII subgroup proteins from *Arabidopsis* (RAP2.2, RAP2.12, RAP2.3, HRE2, HRE1), *Oryza* (SUB1B, SUB1A-1, SUB1C, SK1, SK2), *Capsicum* (CaPF1), *Lycopersicon* (JERF3) and *Actinidia* (PSR94738.1). Tree topology was constructed using MEGA6 programs. The numbers above or below the branches indicated the bootstrap values from 1000 replicates. AdRAP2.3 is marked with a black triangle.

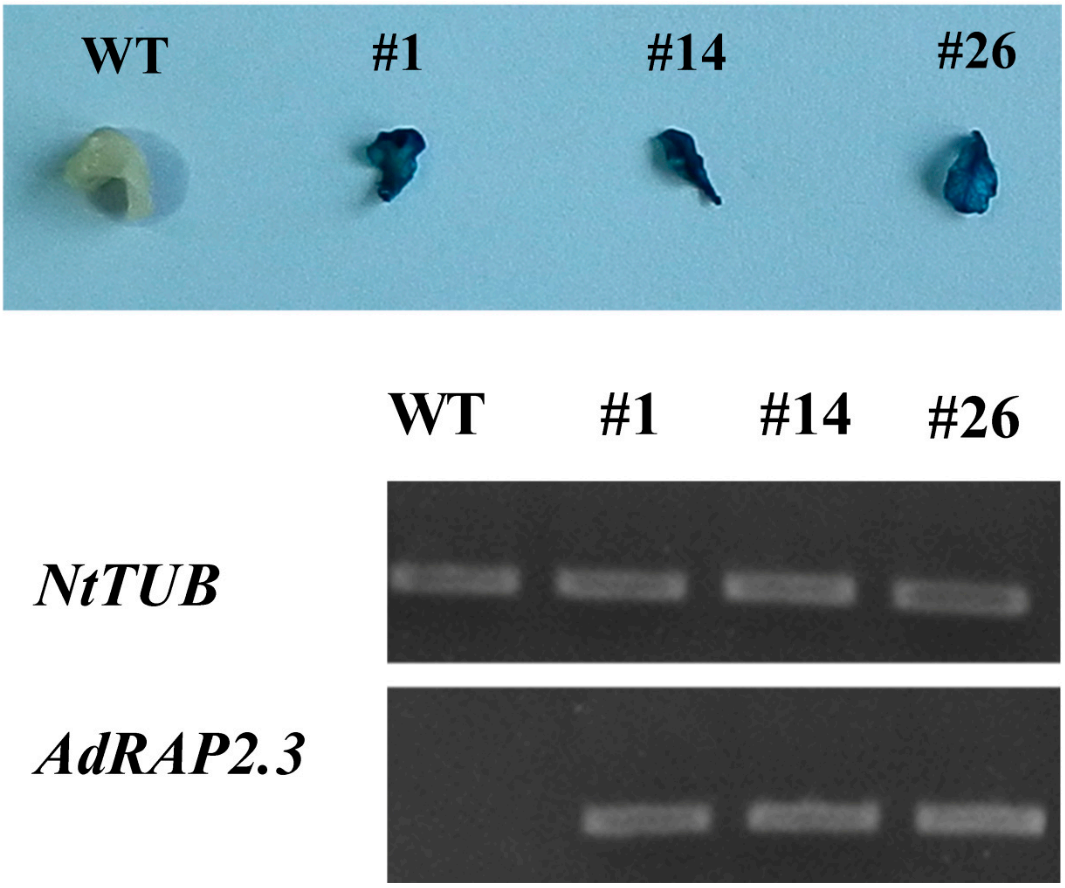

Figure S5. GUS and PCR detection of transgenic tobacco. WT: Wild type; #1, #14, #26: Transgenic tobacco lines.

Table S1. Sequence of primers

Gene name	Primer name	Sequence 5'-3'	Gene ID
AdRAP2.3	F1	CCCGTTCTTCTTATCTTC	
	R1	TATCGTTCCAACTCGGGA	
	F2	AGGAAGCAGCAGGGGAAGAG	
	R2	CAGCACCACAGCCGATGAC	
AdActin	F	TGCATGAGCGATCAAGTTTCAAG	NA
	R	TGTCCCATGTCTGGTTGATGACT	
NtTub	F	AGATGTTCCGTCGTGTCAGTG	EF051136
	R	TGCTTCCTCTTCATCCTCATATCC	
NtPDC	F	GGACTACACTGGTCTTGTGAATGC	XM_016599474
	R	GTGCTTGTTGCTGTTGCTATTGC	
NtADH	F	CGTTCTGATCTTCCTTCTGTCGTC	NM_001325141
	R	CAATTAGTCCTCCATGTTGATGATGC	
NtHB1	F	TGGAGCGTTGATATGAAGAATGCG	XM_016636369
	R	ACGAAAGACATAACCCAACAGGAG	
NtHB2	F	GATCCGTCCATCTCCAAAAGGG	XM_016615748
	R	CGTCTTCGCTCCATTTCTCTCC	
NtPCO1	F	CAATGCGGCTGGGGATTCAAC	XM_016577567
	R	GCGACCTTCAGGATCACAATATGG	
NtPCO2	F	GGTCATCCTACAGTGAGACTAGCC	XM_016660262
	R	AAGAATATCCAGAACCGCACAAGG	

F: Forward primer, R: Reverse primer. AdRAP2.3F1, AdRAP2.3R1 the pair of primer for *AdRAP2.3* gene clone, AdRAP2.3F2, AdRAP2.3R2 the pair of primer for transgenic plant detection and RT-PCR. *AdActin* the reference gene of kiwifruit, *NtTub* the reference gene of tobacco.
